# Reflection on Loving Pedagogy and Students' Engagement in EFL/ESL Classrooms

**DOI:** 10.3389/fpsyg.2021.757697

**Published:** 2021-09-17

**Authors:** Shengxue Zhao, Manman Li

**Affiliations:** ^1^School of Foreign Languages, Bengbu University, Bengbu, China; ^2^School of Foreign Languages, West Anhui University, Lu'an, China

**Keywords:** positive psychology, EFL/ESL, loving pedagogy, students' engagement, emotions

## Abstract

The role of positive emotions in language education has been exponentially approved in the literature. One such emotion which has been mostly neglected in EFL/ESL contexts due to irrational ethical and professional sensitivities is the concept of love. Although love in education highlights a caring environment and relationship which is oriented toward students' feelings and needs, little (if any) research has been done on a loving pedagogy in the context of EFL/ESL. Trying to shed some light on this novel construct, this review article presents the theoretical underpinnings of love, its definitions, dimensions, and positive outcomes in language learning. Moreover, two trends of positive psychology and affective pedagogy are described. Finally, the study presents the possible implications of this line of research for different stakeholders in EFL/ESL domains along with a number of research gaps and future directions for avid scholars in this area.

## Introduction

Language teaching and learning are entangled with one's affects and emotional states (Dewaele and Li, 2020). Emotions play a critical role in academia whose penultimate purpose is to step beyond one's academic performance. This affective turn in education has flourished with novel ideas pinpointed in *Positive Psychology* (PP) which highlights the significance of positive emotions and the way one can thrive (Gabryś-Barker and Gałajda, 2016; Seligman, 2018; Dewaele et al., 2019; Budzińska and Majchrzak, 2021; Wang et al., 2021). One of such positive emotions that is believed to be a basic human need is love (Maslow, 2013). It is a natural desire which affects students' social and emotional development (Dowling, 2014). It also has the power to motivate learners to pursue knowledge, unite them with teachers, and push the existing level of knowledge forwards (Cho, 2005). Love is the cornerstone of meaningful and successful instruction and its absence makes education a simple *training*.

Although love has been sufficiently written about in psychology and philosophy since sixteenth century, its application to education, in general, and language teaching, in particular, has recently gained momentum (Wilkinson and Kaukko, 2020). Love manifests itself through a caring environment, mutual rapport between the teacher and students, and classroom practices. Hence, it can influence students' motivation, well-being, engagement, achievement, and interpersonal communication skills. Nevertheless, the nexus of love and pedagogy has long been a discomfort venue among L2 practitioners as they fear trespassing ethical and professional boundaries in education. Trying to break the ice, in the new millennium, some scholars proposed concepts of *pedagogical love* and a *loving pedagogy* in language education (Loreman, 2011; Yin et al., 2019; Wang et al., 2021). Simply, the notions refer to the care, sensitivity, and empathy that teachers have toward their students' needs, learning experiences, and development (Yin et al., 2019).

Research corroborates that adopting a loving pedagogy increases learners' autonomy, learning motivation, agency, self-esteem, critical-thinking, positive interpersonal behaviors, and academic performance (Grimmer, 2021; Xie and Derakhshan, 2021). However, in EFL contexts, the role of loving pedagogy in promoting students' level of engagement in the classroom and task accomplishment has largely been kept under the carpet. Against this backdrop, the present study aimed to reflect on the possible contributions of loving pedagogy as a novel concept in L2 education to EFL students' engagement.

## Theoretical Background

### Positive Psychology and Affective Pedagogy

As cognitive perspectives gave momentum to the emotional aspects of L2 education, the linkage of emotions and inner states to success and achievement witnessed a scholarly boom (Prior, 2019). The centrality of emotions in education is best addressed in *positive psychology* (PP) school. PP underscores how people can thrive and have happier lives (MacIntyre et al., 2019). Instead of dwelling on negative emotions, PP inspires the practitioners to capitalize on the power of positive emotions like joy, interest, passion, resilience, optimism, and the like to inhibit the negative stressors. PP examines how individuals can flourish and be happier by focusing on positive emotions like joy, hope, passion, love, resilience, optimism, enjoyment, and the like instead of negative feelings (Seligman, 2007; Pishghadam et al., 2021; Wang et al., 2021). According to MacIntyre and Mercer (2014), PP rests on three pillars of *positive subjective experience* (emotions), *positive individual traits* (individual characteristics), and *positive institutions* (contexts).

On the other hand, affective pedagogy (AP) is a way of teaching designed to arouse certain emotional states (Ainsworth and Bell, 2020). It is a part of a wider paradigmatic shift in education called *the affective turn* (Clough, 2007), which highlights the prominence of affective experiences and states in learning. AP foregrounds the development of psychopedagogies to increase students' inner states which are preconditions for their academic achievement (Williamson, 2016). It concerns both emotions and learning outcomes and is characterized by intimacy and ‘dramatic friendships' between the teacher and students (Patience, 2008).

### Conceptualizations of Love and a Loving Pedagogy

Pedagogical love has an influential role in developing students' emotional status, social ability, personality, and mental health (Yin et al., 2019). It has been conceptualized differently since sixteenth century. Love is a multi-faceted concept that is considered as a strong learning motivator, a quality of good education, the basis of classroom interactions, and a natural need (Loreman, 2011). Moreover, it has been defined through psychological, religious, and philosophical perspectives. In a triangular theory of love, Sternberg (1986) conceptualized love to include three elements; *intimacy, passion*, and *decision/ commitment*. A perfect relationship in education, then, is one in which these three elements align together. Religious views, however, come at love differently. They mostly regard love as divine and a feature of God. It is a vital ingredient in the rapport between the teacher and students which makes learning worthy (Loreman, 2011). Philosophically, love has been described by Plato and Aristotle as a search for beauty which can be *eros* (sextual, passionate love), *philia* (love of friends and equals), or *agape* (love of mankind).

In education, love is represented *via* “loving pedagogy,” a recent term developed by Loreman (2011) and Wang et al. (2021). It is a positive learning experience which is momentous for practitioners. It includes 9 emotional concepts of *passion, kindness, empathy, intimacy, bonding, sacrifice, forgiveness, acceptance*, and *community*. Such education is characterized by a caring contexts sensitive toward students' needs, expectations, and emotions. A loving pedagogy is an amalgamation of the mentioned concepts between the teacher and students and is external to us but influences our spirit (Loreman, 2011). It must be the main goal of education as it can nourish students' socio-emotional states, achievement, involvement, and interpersonal skills.

### Student's Engagement in L2 Education

Student engagement is a significant concept in all educational systems as it can generate energy, investment, and success in academia (Eccles, 2016). It refers to students' amount of involvement in the classroom during instructional activities (Skinner and Pitzer, 2012). It is a dynamic variable that is affected by many internal and external factors (Guilloteaux, 2016). Engagement is an explicit sign of intrinsic motivation which has been given different conceptualizations. However, it is substantiated that the concept is multi-dimensional including behavioral, emotional (or affective), cognitive, agentic, academic, and social dimensions (Reschly and Christenson, 2012; Hiver et al., 2020, 2021). *Behavioral engagement* concerns students' active involvement in the classroom activities (e.g., listening, doing tasks, asking questions, and doing the assignments), while *emotional engagement* highlights students' internal states and their affective reactions. Moreover, *cognitive engagement* refers to students' psychological investment in learning and employing complicated learning strategies during a task. *Agentic engagement* is related to students' contribution to learning-teaching quality enhancement. Likewise, *academic engagement* is a student's psychological and behavioral efforts to acquire the knowledge and skills of an academic work. Finally, *social engagement* concerns students' involvement in a range of tasks intended to stimulate their social interaction and problem-solving (DeVito, 2016).

Research reveals that student's engagement can strongly predict different academic outcomes including achievement, psycho-social adjustment, resilience, effective learning, and academic success and can be taught and improved (Eccles, 2016; Jang et al., 2016). Due to its malleable nature, student's engagement is influenced by *phenomenological factors* (task type/difficulty/value, ability level, and culture), *demographic factors* (e.g., age, gender, background, and academic level), and *instructional factors* (e.g., teachers' practices, behaviors, motivation to teach, expertise, and teaching style) (Guilloteaux, 2016). Furthermore, as put by Quin (2017), the teacher-student rapport and classroom climate directly affect students' engagement. Hence, EFL teachers' caring behaviors and the adoption of a pedagogy of love play a critical role in producing, maintaining, and improving engagement among their students (DeVito, 2016).

## Implications, Gaps, and Future Directions

This review article can bring about valuable insights for EFL/ESL teachers, students, teacher educators, materials developers, and L2 researchers. It is deemed significant for EFL/ESL teachers in that they can identify the criticality and power of a caring, friendly, and love-induced educational context in improving many aspects of teaching and learning processes. Teaching is by no means an emotion-free profession but laden with psychological and emotional states which considerably affect the teaching-learning cycle. As love is a meta-construct including at least 9 interrelated concepts mentioned earlier, by establishing a pedagogy of love, teachers can cause many positive outcomes in L2 education. As for students, this study can cast more light on the role of emotions and classroom rapport on students' motivation, engagement, and academic success. When it comes to EFL teaching context in China, it seems that only passing knowledge onto students is far from sufficient for EFL teachers in their language education classes, and they should also try their best to establish teacher-student rapport so that a harmonious class environment can be realized. Also, Chinese EFL teachers in their teaching practice of Higher learning should not only leave their classes without any further emotional contact with their students. For example, some freshers have just stepped into universities from their senior high schools. In most cases, they cannot get used to the new learning environment and they may be encountered with a variety of learning problems, such as learning strategies, learning resources as well as learning methods. When they have such problems, they may suffer from language learning anxiety. Another fact that needs to be noted is that students come from different provinces and they are assessed by different measures. For instance, some students may not have been tested listening competence although all of them need to take Gaokao (College Entrance Examination in China) before they are admitted into institutes of higher learning. However, due to the washback effect of tests on teaching syllabus, the majority of the stakeholders may not take listening instruction seriously. That is, listening competence is not tested, so it will not be trained and those coming from these provinces may have listening anxiety. If those students' EFL teachers can communicate with them, listen to their voices and provide them some constructive suggestions, those students faced with listening difficulties may feel less anxious while they are having their English classes. Finally, successful learning can take place. Hence, EFL/ESL students can work toward an education that goes beyond the simple pursue of learning outcomes and make efforts to constitute an environment based on love and respect.

Teacher trainers can work on teachers' abilities and skills to deal with students' emotions and teach them practical strategies to establish a pedagogy of love *via* workshops and training programs. Likewise, materials developers can use this review to design tasks and textbooks in which students' emotions are reflected along with pedagogical objectives. There must be a place for love in materials so that teachers and students get involved in the books and their activities. Finally, researchers can benefit from this study in that they can run similar studies on the construct of loving pedagogy and fill the existing gaps in this area. As empirical studies on loving pedagogy are rare, L2 researchers can conduct studies on this notion in light of interpersonal communication factors like care, clarity, credibility, immediacy, and confirmation. They can also cross-culturally compare the dimensions of love in different cultures to see if there can be added other dimensions or not. Correlational studies on PP factors such as resilience, enjoyment, success, buoyancy, optimism and the like are also suggested. Moreover, future scholars can use case and longitudinal studies to unpack the developmental trajectories of a loving pedagogy in different contexts and their possible outcomes for different stakeholders. Finally, owing to its dynamism, love can be measured through different research instruments. Hence, avid researchers are recommended to design and validate questionnaires and surveys which can best measure this influential factor in language education.

## Author Contributions

SZ: conceptualizing and writing up the first draft. ML: revising and editing the language. Both authors contributed to the article and approved the submitted version.

## Funding

This study was sponsored by 2019 the Construction of the Process Evaluation System of Internet Plus College English Teaching in provincial quality engineering projects of Higher Institutions (Grant No. 2019jyxm0469); MOOC Demonstrating Project (College English I➁) in Anhui provincial quality engineering project (Grant No. 2018mooc147); 2020 provincial quality engineering demonstration center project of colleges and universities Ideological and political construction demonstration center of college english course of school of foreign languages of Bengbu University (Grant No. 2020szzx25); and Anhui Provincial Education Department in 2017 Major Project of Humanistic and Social Science in Anhui Provincial Universities, Anhui, China (Grant No. SK2017A0633).

## Conflict of Interest

The authors declare that the research was conducted in the absence of any commercial or financial relationships that could be construed as a potential conflict of interest.

## Publisher's Note

All claims expressed in this article are solely those of the authors and do not necessarily represent those of their affiliated organizations, or those of the publisher, the editors and the reviewers. Any product that may be evaluated in this article, or claim that may be made by its manufacturer, is not guaranteed or endorsed by the publisher.
